# A novel diagnostic method for malaria using loop-mediated isothermal amplification (LAMP) and MinION™ nanopore sequencer

**DOI:** 10.1186/s12879-017-2718-9

**Published:** 2017-09-13

**Authors:** Kazuo Imai, Norihito Tarumoto, Kazuhisa Misawa, Lucky Ronald Runtuwene, Jun Sakai, Kyoko Hayashida, Yuki Eshita, Ryuichiro Maeda, Josef Tuda, Takashi Murakami, Shigefumi Maesaki, Yutaka Suzuki, Junya Yamagishi, Takuya Maeda

**Affiliations:** 1 Division of Infectious Diseases and Pulmonary Medicine, Department of Internal Medicine, National Defense Medical College, 3-2 Namiki, Tokorozawa, Saitama, 359-8513 Japan; 2 Department of Infectious Disease and Infection Control, Saitama Medical University, 38 Morohongo, Moroyama-machi, Iruma-gun, Saitama, 350-0495 Japan; 3 Center for Clinical Infectious Diseases and Research, Saitama Medical University, 38 Morohongo, Moroyama-machi, Iruma-gun, Saitama, 350-0495 Japan; 4 Department of Computational Biology and Medical Science, Graduate School of Frontier Sciences, The University of Tokyo, 5-1-5 Kashiwanoha, Kashiwa, Chiba, 277-8562 Japan; 5 Research Center for Zoonosis Control, Hokkaido University, North 20, West 10 Kita-ku, Sapporo, Hokkaido 001-0020 Japan; 6 Faculty of Medicine, Oita University, 1-1 Hasama-machi, Yufu, Oita 879-5593 Japan; 7 Department of Medical Entomology, Faculty of Tropical Medicine, Mahidol University, 420/6 Ratchawithi Road, Thung Phaya, Ratchathewi, Bangkok, 10400 Thailand; 8 Research Institute for Microbial Diseases, Osaka University, 3-1 Yamadaoka, Suita, Osaka, 565-0871 Japan; 9 Division of Biomedical Sciences, Department of Basic Veterinary Medicine, Obihiro University of Agriculture and Veterinary Medicine, 2-11 Inada-cho, Obihiro, Hokkaido 080-8555 Japan; 10 Department of Parasitology, Faculty of Medicine, Sam Ratulangi University, Kampus Unsrat, Manado, Bahu 95115 Indonesia; 11 Department of Microbiology, Saitama Medical University, 38 Morohongo, Moroyama-machi, Iruma-gun, Saitama, 350-0495 Japan; 12 Global Station for Zoonosis Control, GI-CoRE, Hokkaido University, North 20, West 10 Kita-ku, Sapporo, Hokkaido 001-0020 Japan

**Keywords:** Malaria, *Plasmodium ovale curtisi*, *Plasmodium ovale wallikeri*, LAMP, Nanopore sequencer, MinION™, Diagnosis

## Abstract

**Background:**

A simple and accurate molecular diagnostic method for malaria is urgently needed due to the limitations of conventional microscopic examination. In this study, we demonstrate a new diagnostic procedure for human malaria using loop mediated isothermal amplification (LAMP) and the MinION™ nanopore sequencer.

**Methods:**

We generated specific LAMP primers targeting the 18S–rRNA gene of all five human *Plasmodium* species including two *P. ovale* subspecies (*P. falciparum, P. vivax, P. ovale wallikeri, P. ovale curtisi*, *P. knowlesi* and *P. malariae*) and examined human blood samples collected from 63 malaria patients in Indonesia. Additionally, we performed amplicon sequencing of our LAMP products using MinION™ nanopore sequencer to identify each *Plasmodium* species.

**Results:**

Our LAMP method allowed amplification of all targeted 18S–rRNA genes of the reference plasmids with detection limits of 10–100 copies per reaction. Among the 63 clinical samples, 54 and 55 samples were positive by nested PCR and our LAMP method, respectively. Identification of the *Plasmodium* species by LAMP amplicon sequencing analysis using the MinION™ was consistent with the reference plasmid sequences and the results of nested PCR.

**Conclusions:**

Our diagnostic method combined with LAMP and MinION™ could become a simple and accurate tool for the identification of human *Plasmodium* species, even in resource-limited situations.

**Electronic supplementary material:**

The online version of this article (10.1186/s12879-017-2718-9) contains supplementary material, which is available to authorized users.

## Background

Malaria is a severe public health problem prevalent in tropical and subtropical areas worldwide. It is a mosquito-borne infectious disease caused by the following five protozoan parasites: *Plasmodium falciparum*, *P. vivax*, *P. malariae*, *P. ovale* and *P. knowlesi*. There are emerging differences among first-line treatments for these parasites, which are dependent on the *Plasmodium* species and appropriate selection of anti-malarial drugs based on accurate recognition of the pathogens. Thus, it is important to correctly identify the pathogen after the initial diagnosis and before standard empirical chemotherapy commences. The conventional gold standard method of diagnosing malaria involves microscopic examination of Giemsa-stained thick blood smears. This method is inexpensive and easily implemented, even in remote endemic areas. However, microscopy is not as sensitive as molecular diagnostic methods for the identification of species, and both the sensitivity and specificity often depend on the technical skill of the investigator. In fact, compared with molecular diagnostic methods such as polymerase chain reaction (PCR), only half the number of patients with malaria are correctly diagnosed via microscopy in endemic areas [1, 2]. Additionally, some *Plasmodium* species, such as *P. knowlesi* and *P. malariae,* are difficult to distinguish from each other based on morphological features*.* The presence of *P. knowlesi* has most recently been reported in East Asian countries, especially Malaysia [3]. Although the first confirmed case of human *P. knowlesi* infection was reported in 1965 [4], it has now become clear from the results of a retrospective investigation in 2004 using PCR methods that many *P. knowlesi* human infection cases in Malaysia have been misdiagnosed as *P. malariae* by microscopy [5].

In recent years, *P. ovale* has been classified into two distinct subspecies, *P. ovale curtisi* and *P. ovale wallikeri,* based on molecular investigations [6–8], and it has been suggested that there are potential clinical differences such as symptoms and latency duration between the two subspecies [9, 10]. Thus, further epidemiological studies are required to determine the differences in prevalence and distribution between these two subspecies. Nested PCR or semi-nested PCR methods for detecting human *Plasmodium* spp. are widely used, but none can completely detect and simultaneously separate the five species, including the two *P. ovale* subspecies [11–13]. Therefore, a simple molecular diagnostic method corresponding to the latest classification of *Plasmodium* parasites is urgently needed to discriminate between *Plasmodium* spp.

Loop-mediated isothermal amplification (LAMP), which is an innovative nucleic acid amplification (NAA) technique, is regarded as a more rapid, easy-to-perform, and cost-effective procedure than PCR [14]. LAMP methods have already been used for the clinical diagnoses of several infectious diseases, including malaria, and are promising molecular technologies with high validity, even in resource-limited areas [15–37]. However, the following disadvantages of LAMP methods could be problematic: 1) the sequences of LAMP products are difficult to confirm; 2) LAMP primers are difficult to design because of the long nucleotide sequences of the primers; and 3) the primer sets and reaction tubes must be constructed individually, according to the target genes and samples.

The MinION™ nanopore sequencer is a pocket-sized and USB-connected portable real-time sequencer developed by Oxford Nanopore Technologies (ONT; Oxford, UK). The main advantages of the MinION™ are its portability, small platform, long reads, and real-time sequencing. In particular, sequence data can be made available in real time as they are generated and MinION™ can be easily transported and used away from laboratories under various clinical situations [38, 39]. In recent years, MinION™ has already been used to sequence PCR amplicons [40] or amplicons of RT-LAMP procedures [41].

Based on the polymorphisms of 18S–rRNA sequences in the *Plasmodium* species, we demonstrated a rapid and easy-to-perform diagnostic procedure for malaria, which takes advantage of the specific characteristics of the LAMP method as an NAA and MinION™ nanopore sequencer. We propose that this novel diagnostic approach could help to provide accurate diagnoses and appropriate treatments for malaria patients.

## Methods

### Plasmid construction

**Fig. 1:**
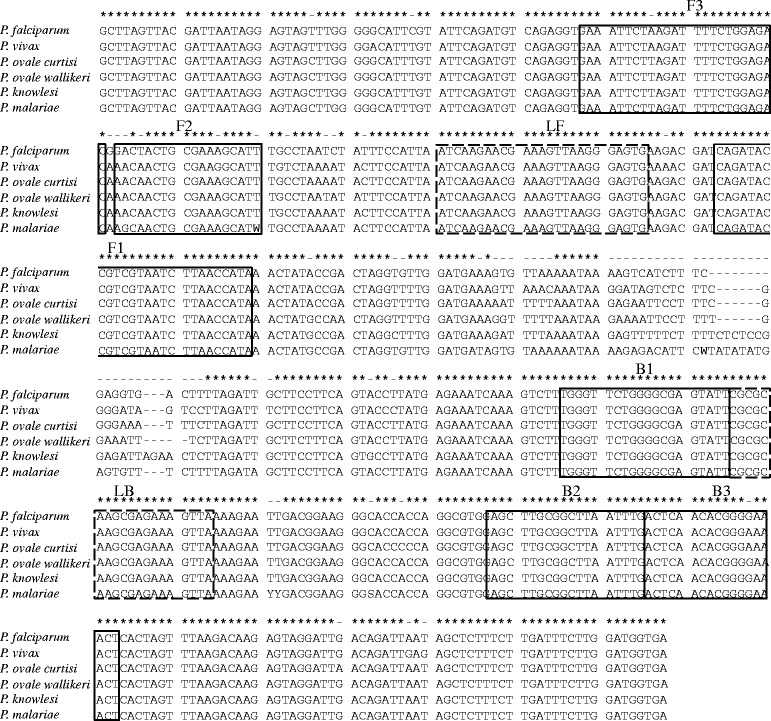
Sequence alignment of 18S–rRNA of human *Plasmodium* parasites and primer locations for LAMP

The templates used for the analytical LAMP reactions were six sets of pEX-A2J1 plasmids harboring partial sequences within the 18S–rRNA of six human *Plasmodium* parasites, *P. falciparum, P. vivax*, *P. ovale curtisi*, *P. ovale wallikeri*, *P. knowlesi* and *P. malariae* (comprising all five human plasmodium species including the two *P. ovale* subspecies) which were constructed by Eurofines Genomics Co., Ltd. (Tokyo, Japan) based on the reference sequences (GenBank accession numbers: *P. falciparum*, HQ283212.1; *P. vivax,* KF018662.1; *P. ovale curtisi*, KF696370.1; *P. ovale wallikeri,* KJ619947.1; *P. knowlesi,* KJ917903.1; and *P. malariae,* LT615260.1). The sequences of the constructed plasmids are summarized in Fig. 1. The plasmids were serially diluted 10-fold and adjusted from 1.0 × 10^1^ copies/μL to 1.0 × 10^6^ copies/μL to create 18S–rRNA standard solutions to determine the detection threshold and specificity of the LAMP reactions.

### Design of LAMP primers

The oligonucleotide LAMP primers for the detection of the five *Plasmodium* species were designed based on the sequences of 18S–rRNA, located on their highly conserved sequences, to contain each species-specific region within the F1-B1 primer pairs. Several candidate primer sets were suggested via online LAMP primer design software (PrimerExplorer 5, http://primerexplorer.jp/index.html; Eiken Chemical, Tokyo, Japan), and further refinement of the design was developed manually based on the criteria described in “A Guide to LAMP primer designing” (https://primerexplorer.jp/e/v4_manual/pdf/PrimerExplorerV4_Manual_1.pdf). Each selected primer sequence is given in Table 1, and their positions are shown in Fig. 1.

**Table 1:** Nucleotide sequences of constructed LAMP primers for 18S–rRNA

Primer	Sequence (5′ - 3′)	Length
F3	GAAATTCTAAGATTTTCTGGAGAC	24
B3	AGTTTTCCCGTGTTGAGT	18
FIP	TATGGTTAAGATTACGACGGTATCTG-RCAACTGCGAAGGCATW	43
BIP	TGGGTTCTGGGGCGAGTATT-CAAATTAAGCCGCAAGCT	38
LF	CACTCCCTTAACTTTCGTTCTTGAT	25
LB	CGCGCAAGCGAGAAAGTTA	19

F3 and B3, outer primers; FIP and BIP, inner primers; LF and LB, loop primers

The FIP primer consisted of F2 and the complementary strand of F1 (F1c)

The BIP primer consisted of B2 and the complementary strand of B1 (B1c)

### LAMP reactions

The reactions were performed using a Loopamp DNA Amplification Reagent Kit (Eiken Chemical). Detection of the LAMP amplicons was performed by real-time measurements of turbidity using Loopamp EXIA (Eiken Chemical) and visual observation of color changes with the naked eye under natural light. The final reaction volume was 25 μL, comprising 80 pmol of the FIP primer and 40 pmol of the BIP primer, 20 pmol of each loop primer, 5 pmol of F3 and B3 primers, 1 μL of the provided *Bst* DNA polymerase, reaction buffer (20 mM Tris-HCl, 10 mM KCl, 8 mM MgSO_4_, 10 mM (NH_4_)_2_SO_4_, 0.1% Tween 20, 0.8 M betaine, and 1.4 mM each dNTP), and 1 μL of plasmid DNA or 1–8 μL of genomic DNA samples. Reactions were carried out at 62 °C, 63 °C, 64 °C, and 65 °C in duplicate to find the optimum temperature for LAMP amplification.

### Detection limit and specificity of the LAMP assay

Ten-fold serial dilutions of each plasmid DNA, ranging from 1 × 10^1^ to 1 × 10^5^ copies/μL, were obtained and stored in duplicate at −20 °C until use, to determine the detection limit of the LAMP assay. Additionally, to evaluate the specificity of the test, we examined seven kinds of protozoa (*Toxoplasma gondii*, *Cryptosporidium parvum*, *Giardia intestinalis*, *Entamoeba histolytica*, *Leishmania donovani*, *Trypanosoma brucei rhodesiense*, and *Trypanosoma cruzi*) isolated by the National BioResource Project (http://www.nbrp.jp/).

### Clinical samples and ethics

Clinical samples were obtained from Sam Ratulangi University in Manado and Bitung, North Sulawesi, Indonesia, from August to December 2010. All patients were diagnosed based on the symptoms of malaria (fever, chills, sweats, fatigue, headache, nausea, vomiting, and general malaise) and blood smears stained with Giemsa stain by medical staff at the hospital. Peripheral blood was collected by FTA Elute cards (GE Healthcare Life Sciences, Little Chalfont, UK) from each patient and stored at room temperature. All samples were confirmed as harboring *Plasmodium* species by species-specific nested-PCR assay targeting the 18S rRNA gene to identify the five human *Plasmodium* species, according to previously reported protocols [42, 43].

Both the design and protocol of this study conformed to the Helsinki Declaration and were approved by the Institutional Ethics Committee. All samples were collected after obtaining written informed consent. The methods of collection and analysis of the human samples were approved and cleared by the Institutional Ethical Review Board of Sam Ratulangi University and the University of Tokyo (approval number 10–49).

### DNA extraction from clinical samples

Genomic DNA was extracted by following the manufacturer’s instructions for FTA Elute cards. Briefly, each disk with a diameter of 3.0 mm was cut from the blood spot areas and washed three times with 200 μL of distilled water. DNA was eluted in 30 μL of TE buffer (10 mM Tris-HCl, 0.1 mM EDTA, pH 8.0) with a heat block at 95 °C for 30 min. Finally, 1.0 μL of supernatant was used as template DNA for the LAMP reactions. If no amplification product was observed, 8 μL of supernatant was also used as template DNA, and we confirmed the final diagnosis by LAMP.

### Sequencing of LAMP products by MinION™

We performed sequencing of the amplicons obtained by LAMP with the MinION™ nanopore sequencer to identify the *Plasmodium* species. Six amplicons of plasmid DNA harboring the partial sequences of 18S–rRNA of each *Plasmodium* parasite from LAMP were used to validate the performance of MinION™. We prepared the DNA library for MinION™ by using the Ligation Sequencing Kit 2D (R9.4) (ONT, Oxford, UK) for each LAMP amplicon. Amplicons (each 2 μg) were processed for end-repair using the NEBNext Ultra™ II End Repair/dA-Tailing Module (New England Biolabs, Ipswich, MA), followed by purification with Agencourt AMPure XP beads (Beckman Coulter, Brea, CA). Each end-prepped DNA was subsequently subjected to adapter ligation with Blunt/TA Ligase Master Mix (New England Biolabs) to obtain the final DNA library following the ONT protocol of the Ligation Sequencing Kit 2D R9.4 version (SQK-LSK 208). Dynabeads MyOne™ Streptavidin C1 (Invitrogen, Carlsbad, CA) was used to elute the library in the pre-sequencing mix. After the MinION™ Platform QC run, the DNA library was loaded into the MinION™ flow cell (FLO-MIN 105 of 106 R9 version) and the standard 48-h sequencing protocol was initiated using the MinKNOW ONT software.

We used the Rapid Barcoding Sequencing Kit (SQK-RBK001) (ONT) for multiplex sequencing of LAMP amplicons from clinical samples, and thus were able to simultaneously sequence 12 samples on a single flow cell. Each LAMP amplicon (derived from one clinical sample) was purified by using Agencourt AMPure XP beads; 200 ng of each LAMP amplicon was processed for MinION™ barcoding and library preparation. Finally, a mixture of 12 barcoded DNA libraries was loaded into another MinION™ flow cell (FLO-MIN 105 of 106 R9 Version or FLO*-*MIN107 R9.4 version) and the standard 48-h 1D sequencing protocol was initiated using the MinKNOW ONT software.

### Data analysis

Data analysis was performed according to the protocol described in previous reports [44–46]. Raw read data were obtained by the standard 48-h sequencing protocol in the MinKNOW software (v1.1.21) and base-called via the ‘2D Basecalling plus Barcoding’ or ‘1D Basecalling plus Barcoding’ workflow (v1.25) of Metrichor (v2.43.1). FASTQ sequences of 2D or 1D pass reads were extracted using Poretools (v0.6.0). 2D or 1D pass data were mapped by BWA-MEM (v0.7.15) using six sequences of *Plasmodium* 18S–rRNA between a pair of LAMP primers, F1c (5′-CAGATACCGTCGTAATCTTAACCATA-3′) and B1c (5′-TGGGTTCTGGGGCGAGTATT-3′). Mapped data were applied in IGVsoftware (v2.3.80) after filtering with mapQuality 60 by BamTools (v 2.3.0). Based on the mapped data, several mapped reads at each of the sequences of *Plasmodium* 18S–rRNA per total number of mapped reads >0.2 were selected as a “consensus” for each sample. Consensus alignment was obtained using SAMtools (v1.4) and BCFtools (v1.4), following BlastN (v2.5.0) for queries against the database nucleotide collection (nr/nt).

## Results

### Detection limit and specificity of LAMP

**Fig. 2:**
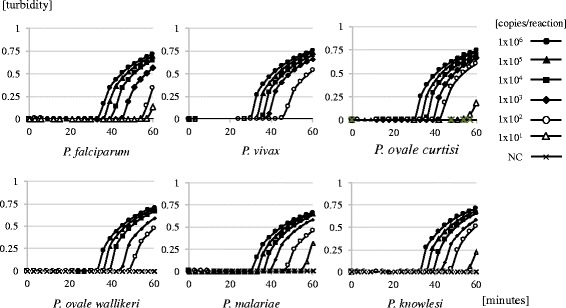
Detection limit of LAMP for the six human *Plasmodium* species with 10-fold serial dilutions of each plasmid DNA

To determine the lower detection limit, 10-fold serial dilutions of each plasmid DNA were amplified and the optimal temperature observed was 62 °C. The results of real-time turbidity detection with LA-200 and amplification of the target DNA are indicated by the rising curve (Fig. 2). The minimum amounts of plasmid DNA on real-time turbidities with LA-200 were 1.0 × 10^1^/reaction (*P. falciparum*, *P. ovale curtisi*, *P. knowlesi, P. malariae*) and 1.0 × 10^2^/reaction (*P. vivax*, *P. ovale wallikeri*) within 60 min (Fig. 2). Furthermore, these results resembled those detected visually based on color changes within 60 min with the naked eye (data not shown). Additionally, 1.0 ng of the genomic DNA, derived from seven kinds of human protozoan parasites, was not completely amplified by our LAMP method (data not shown).

### Identification of plasmodium species by LAMP assay and MinION™

Table 2 shows the results obtained by MinION™ sequencing for the identification of *Plasmodium* species. LAMP amplicons targeting 18S–rRNA from six plasmid DNA molecules containing each species-specific 18S–rRNA were sequenced by MinION™. The *Plasmodium* species identified by MinION™ sequencing were consistent with the sequencing data of the constructed plasmids and the highest homology of reference sequences for *Plasmodium* parasite sequences from the NCBI gene databases.

**Table 2:** Summary of the MinION™ sequencing of LAMP amplicons

Samples	Mapped reads	Aligned bases	Mean coverage	% Identity	Identification of species with MinION™
Plasmids					
*P. falciparum*	97,290	7,321,826	91,522.825	100	*P. falciparum*
*P. vivax*	105,097	8,164,884	99,571.7561	100	*P. vivax*
*P. ovale curtisi*	58,084	4,963,139	54,539.98901	100	*P. ovale curtisi*
*P. ovale wallikeri*	1054	77,098	963.725	100	*P. ovale wallikeri*
*P. knowlesi*	3254	244,871	2986.231707	100	*P. knowlesi*
*P. malariae*	627	51,135	581.0795455	100	*P. malariae*

*Clinical evaluation of LAMP assay and MinION™*

The results of nested PCR and our LAMP method combined with the MinION™ sequencer are summarized in Table 3 and Additional file 1: Table S1. A total of 63 blood samples were obtained from patients with suspected malaria in a clinical setting and parasitemia was confirmed in 54 samples by nested-PCR assay (36 samples had *P. falciparum*, 17 had *P. vivax,* and 1 had a combination of *P. falciparum* and *P. vivax*). All these samples were found to be positive by our LAMP method, and the results of species identification by MinION™ sequencing were consistent with those of nested PCR. Our LAMP method confirmed the negative status of 8 of the 9 negative samples identified by nested PCR. One sample was found to be positive by our LAMP method and subsequent data analysis of MinION™ sequences identified *P. falciparum*.

**Table 3:** Summary of the results of nested PCR and our LAMP method combined with MinION™ sequencer

		Sequence confirmation using MinION™ sequencer							
		*P.f.*	*P.v.*	*P.f./P.v.*	*P.o.w.*	*P.o.c.*	*P.k.*	*P.m.*	Neg
Nested PCR	*P.f.* (*n* = 36)	36	0	0	0	0	0	0	0
	*P.v.* (*n* = 17)	0	17	0	0	0	0	0	0
	*P.f.*/*P.v.* (n = 1)	0	0	1	0	0	0	0	0
	*P.o.* (*n* = 0)	0	0	0	0	0	0	0	0
	*P.k* (n = 0)	0	0	0	0	0	0	0	0
	*P.m.* (n = 0)	0	0	0	0	0	0	0	0
	Neg (*n* = 9)	1	0	0	0	0	0	0	8
	Total (*n* = 63)	37	17	1	0	0	0	0	8

*P.f.*, *P. falciparum; P.v. P. vivax; P.o.*, *P. ovale; P.o.w.*, *P. ovale wallikeri; P.o.c.*, *P. ovale crutisi; P.k.*, *P. knowlesi; P.m.*, *P. malariae;* Neg, negative

## Discussion

In this study, we constructed a novel tool for the identification of six *Plasmodium* parasites, including two *P. ovale* subspecies, using MinION™ portable sequencer and the LAMP method. We demonstrated that the method can achieve a comprehensive diagnosis of malaria even in resource-limited endemic regions. In particular, the LAMP method employs a set of four specially designed primers that recognize six distinct sequences on the target DNA, and relies on an auto-cycling procedure under isothermal conditions. In addition, our method enabled rapid library preparation (within 30 min), with multiplex sequencing and streaming analysis of real-time sequencing data via the Rapid Barcoding Sequencing Kit. Therefore, our LAMP method combined with the MinION™ sequencer would be convenient to use in various clinical settings.

Recently, Loopamp™ MALARIA Pan/Pf Detection Kit (Eiken Chemical) has become commercially available for the diagnosis of malaria by the LAMP method. This LAMP kit has good specificity and a lower detection limit of 25 parasites/μL that exceeds the detection sensitivity of rapid diagnostic test (RDT) kits for *P. falciparum*-specific histidine-rich protein II, which often yields false-negative results when the parasite density is low, especially when asexual parasitemia is below 200 parasites/μL [47, 48]. RDTs are the most convenient tools for the diagnosis of *P. falciparum* parasites as point-of-care testing in the endemic field because they do not require electricity or special equipment. However, *plasmodium* lactate dehydrogenase or aldolase-based RDT kits cannot detect non-*P. falciparum* adequately because of their low sensitivity [49–53]. In this study, the detection limit of our LAMP method for all *Plasmodium* species was 1.0 × 10^1^ to 1.0 × 10^2^ copies/reaction (1–8 μL of sample DNA could be loaded per reaction); therefore, the theoretical detection limit of the LAMP assay was equivalent to that of the previously described Loopamp™ MALARIA Pan/Pf Detection Kit. Additionally, our LAMP method combined with the MinION™ sequencer has the following advantages: (a) equally high detection sensitivity for all *Plasmodium* species using our protocol, (b) multiplex sequencing and streaming analysis of real-time sequencing data [54] can be achieved, and (c) detection of mixed parasite infections is possible.

Nested-PCR and real-time PCR methods for malaria have previously demonstrated higher sensitivity and specificity than microscopy and RDTs [55]. However, the analytical positive and negative rate of our LAMP method was equivalent to that of nested-PCR methods, showing its potential as a highly practical diagnostic tool. Among the 63 clinical samples, the results of only 1 were discordant between nested PCR and our LAMP method; nested PCR gave a negative result while our LAMP method gave a positive result. The species was identified as *P. falciparum* based on the LAMP amplicon sequencing by MinION™. It is thought that the parasite density was very low in this sample and below the detection limit of nested PCR. Furthermore, our technology has the advantage of being able to comprehensively and simultaneously distinguish and identify six human malaria parasites including two *P. ovale* subspecies, whereas both PCR-based protocols and RDTs cannot comprehensively detect and separate all species. Adams et al. reported that the LAMP primer set does not require stringent reaction conditions, and the reaction is successful at a broad temperature range of 60–66 °C. They demonstrated that the set should be suitable for use in the LAMP assay even in water baths where the temperatures may fluctuate widely [56]. However, nested-PCR and real-time PCR methods remain unsuitable for routine diagnostic methods in hospitals and field clinics in resource-limited endemic regions, because they require expensive devices such as thermal cyclers or real-time PCR systems for accurate temperature control.

Techniques for genotyping gene polymorphisms without sequence analysis have been previously developed using the high specificity of the LAMP assay, including LAMP with FIP and/or BIP primers, which were designed to contain a single-nucleotide polymorphism at each 5′ end and prevent the strands from annealing completely with the mutant allele to form the essential dumbbell-like structure [57–59]. Alternatively, peptide nucleic acid-locked nucleic acid mediated LAMP and real-time LAMP combined with fluorescent probes and melting or annealing curve analyses have been reported for genotyping polymorphisms [60, 61]. However, the design of LAMP primers for the above-mentioned protocol is not only quite difficult and limited, but is less sensitive than conventional LAMP assays as a result. In contrast, by our methods with MinION™ sequencing, LAMP primer design for genotyping polymorphisms for the absolute identification of *Plasmodium* parasite species is possible without limitations and could be applicable to simple mutation analysis or genotyping as the next step.

The MinION™ has been widely used to sequence various pathogens in many clinical situations because of its palm-sized platform, USB-3 interfaced equipment, real-time accessibility of sequenced data, and very low initial costs. In addition, the MinION™ can read multiplexed samples (up to 96) in one flow cell via the MinION™-specific barcoding primers (PCR Barcoding Kit 96 (R9), ONT); therefore, the running costs and time taken for analyses are reduced [62]. However, we do not have clinical samples to confirm the detection limits of *P. ovale*, *P. knowlesi,* and *P. malariae*; thus, we have not demonstrated the utility of our method for detecting these *Plasmodium* infections in clinical samples. An accurate evaluation of the sensitivity and specificity of our method requires further investigation in a clinical setting.

## Conclusion

In this study, we demonstrated an innovative diagnostic technology for *Plasmodium* infection that is rapid, simple, and highly sensitive using the LAMP assay combined with MinION™ nanopore sequencer. Our method could contribute to the provision of appropriate treatment for malaria and could also be applied to further analyses of sequence polymorphisms or genotyping. To refine this procedure, the next step will be to conduct a clinical evaluation to ensure sufficient and consistent sensitivity and specificity in resource-limited endemic regions.

## Additional file

Additional file 1: The result of nested-PCR, LAMP method and MinIONTM sequencing of LAMP amplicons. (XLSX 19 kb)

## Abbreviations

LAMP: loop mediated isothermal amplification
NAA: Nucleic acid amplification
PCR: Polymerase chain reaction
RDT: Rapid diagnostic test

## References

[1] Cheng Q, Cunningham J, Gatton ML (2015). Systematic review of sub-microscopic *P. vivax* infections: prevalence and determining factors. PLoS Negl Trop Dis.

[2] Okell LC, Ghani AC, Lyons E, Drakeley CJ (2009). Submicroscopic infection in *Plasmodium falciparum*-endemic populations: a systematic review and meta-analysis. J Infect Dis.

[3] Singh B, Daneshvar C (2013). Human infections and detection of *Plasmodium knowlesi*. Clin Microbiol Rev.

[4] Chin W, Contacos PG, Coatney GR, Kimball HR (1965). A naturally acquited quotidian-type malaria in man transferable to monkeys. Science.

[5] Singh B, Kim Sung L, Matusop A, Radhakrishnan A, Shamsul SS, Cox-Singh J (2004). A large focus of naturally acquired *Plasmodium knowlesi* infections in human beings. Lancet.

[6] Win TT, Jalloh A, Tantular IS, Tsuboi T, Ferreira MU, Kimura M (2004). Molecular analysis of *Plasmodium ovale* variants. Emerg Infect Dis.

[7] Sutherland CJ, Tanomsing N, Nolder D, Oguike M, Jennison C, Pukrittayakamee S (2010). Two nonrecombining sympatric forms of the human malaria parasite *Plasmodium ovale* occur globally. J Infect Dis.

[8] Oguike MC, Betson M, Burke M, Nolder D, Stothard JR, Kleinschmidt I (2011). *Plasmodium ovale curtisi* and *Plasmodium ovale wallikeri* circulate simultaneously in African communities. Int J Parasitol.

[9] Nolder D, Oguike MC, Maxwell-Scott H, Niyazi HA, Smith V, Chiodini PL, et al. An observational study of malaria in British travellers: *Plasmodium ovale wallikeri* and *Plasmodium ovale curtisi* differ significantly in the duration of latency. BMJ Open. 2013; 10.1136/bmjopen-2013-002711. 10.1136/bmjopen-2013-002711 PMC3657643 23793668

[10] Rojo-Marcos G, Rubio-Munoz JM, Ramirez-Olivencia G, Garcia-Bujalance S, Elcuaz-Romano R, Diaz-Menendez M (2014). Comparison of imported *Plasmodium ovale curtisi* and *P. ovale wallikeri* infections among patients in Spain, 2005-2011. Emerg Infect Dis.

[11] Snounou G, Singh B (2002). Nested PCR analysis of *Plasmodium* parasites. Methods Mol Med.

[12] Snounou G, Viriyakosol S, Zhu XP, Jarra W, Pinheiro L, do Rosario VE (1993). High sensitivity of detection of human malaria parasites by the use of nested polymerase chain reaction. Mol Biochem Parasitol.

[13] Singh B, Bobogare A, Cox-Singh J, Snounou G, Abdullah MS, Rahman HA (1999). A genus- and species-specific nested polymerase chain reaction malaria detection assay for epidemiologic studies. Am J Trop Med Hyg..

[14] Mori Y, Notomi T (2009). Loop-mediated isothermal amplification (LAMP): a rapid, accurate, and cost-effective diagnostic method for infectious diseases. J Infect Chemother.

[15] Cuadros J, Perez-Tanoira R, Prieto-Perez L, Martin-Martin I, Berzosa P, Gonzalez V (2015). Field evaluation of malaria microscopy, rapid malaria tests and loop-mediated isothermal amplification in a rural hospital in south western Ethiopia. PLoS One.

[16] Lau YL, Lai MY, Fong MY, Jelip J, Mahmud R (2016). Loop-mediated isothermal amplification assay for identification of five human *Plasmodium* species in Malaysia. Am J Trop Med Hyg..

[17] Lucchi NW, Ljolje D, Silva-Flannery L, Udhayakumar V (2016). Use of malachite green-loop mediated isothermal amplification for detection of *Plasmodium* spp. parasites. PLoS One.

[18] Modak SS, Barber CA, Geva E, Abrams WR, Malamud D, Ongagna YS (2016). Rapid point-of-care isothermal amplification assay for the detection of malaria without nucleic acid purification. Infect Dis (Auckl).

[19] Britton S, Cheng Q, Grigg MJ, Poole CB, Pasay C, William T (2016). Sensitive detection of *Plasmodium vivax* using a high-throughput, colourimetric loop mediated isothermal amplification (HtLAMP) platform: a potential novel tool for malaria elimination. PLoS Negl Trop Dis.

[20] Britton S, Cheng Q, Grigg MJ, William T, Anstey NM, McCarthy JS (2016). A sensitive, colorimetric, high-throughput loop-mediated isothermal amplification assay for the detection of *Plasmodium knowlesi*. Am J Trop Med Hyg..

[21] Rek J, Katrak S, Obasi H, Nayebare P, Katureebe A, Kakande E (2016). Characterizing microscopic and submicroscopic malaria parasitaemia at three sites with varied transmission intensity in Uganda. Malar J.

[22] Adams ER, Gomez MA, Scheske L, Rios R, Marquez R, Cossio A (2014). Sensitive diagnosis of cutaneous leishmaniasis by lesion swab sampling coupled to qPCR. Parasitology.

[23] Patel JC, Oberstaller J, Xayavong M, Narayanan J, DeBarry JD, Srinivasamoorthy G (2013). Real-time loop-mediated isothermal amplification (RealAmp) for the species-specific identification of *Plasmodium vivax*. PLoS One.

[24] Lee PW, Ji DD, Liu CT, Rampao HS, do Rosario VE, Lin IF (2012). Application of loop-mediated isothermal amplification for malaria diagnosis during a follow-up study in Sao Tome. Malar J.

[25] Sirichaisinthop J, Buates S, Watanabe R, Han ET, Suktawonjaroenpon W, Krasaesub S (2011). Evaluation of loop-mediated isothermal amplification (LAMP) for malaria diagnosis in a field setting. Am J Trop Med Hyg..

[26] Lau YL, Fong MY, Mahmud R, Chang PY, Palaeya V, Cheong FW (2011). Specific, sensitive and rapid detection of human *Plasmodium knowlesi* infection by loop-mediated isothermal amplification (LAMP) in blood samples. Malar J.

[27] Tao ZY, Zhou HY, Xia H, Xu S, Zhu HW, Culleton RL (2011). Adaptation of a visualized loop-mediated isothermal amplification technique for field detection of *Plasmodium vivax* infection. Parasit Vectors.

[28] Hopkins H, Gonzalez IJ, Polley SD, Angutoko P, Ategeka J, Asiimwe C (2013). Highly sensitive detection of malaria parasitemia in a malaria-endemic setting: performance of a new loop-mediated isothermal amplification kit in a remote clinic in Uganda. J Infect Dis.

[29] Lucchi NW, Demas A, Narayanan J, Sumari D, Kabanywanyi A, Kachur SP (2010). Real-time fluorescence loop mediated isothermal amplification for the diagnosis of malaria. PLoS One.

[30] Mohon AN, Elahi R, Khan WA, Haque R, Sullivan DJ, Alam MS (2014). A new visually improved and sensitive loop mediated isothermal amplification (LAMP) for diagnosis of symptomatic falciparum malaria. Acta Trop.

[31] Han ET, Watanabe R, Sattabongkot J, Khuntirat B, Sirichaisinthop J, Iriko H (2007). Detection of four *Plasmodium* species by genus- and species-specific loop-mediated isothermal amplification for clinical diagnosis. J Clin Microbiol.

[32] Patel JC, Lucchi NW, Srivastava P, Lin JT, Sug-Aram R, Aruncharus S (2014). Field evaluation of a real-time fluorescence loop-mediated isothermal amplification assay, RealAmp, for the diagnosis of malaria in Thailand and India. J Infect Dis.

[33] Aydin-Schmidt B, Xu W, Gonzalez IJ, Polley SD, Bell D, Shakely D (2014). Loop mediated isothermal amplification (LAMP) accurately detects malaria DNA from filter paper blood samples of low density parasitaemias. PLoS One.

[34] Dinzouna-Boutamba SD, Yang HW, Joo SY, Jeong S, Na BK, Inoue N (2014). The development of loop-mediated isothermal amplification targeting alpha-tubulin DNA for the rapid detection of *Plasmodium vivax*. Malar J.

[35] Cook J, Aydin-Schmidt B, Gonzalez IJ, Bell D, Edlund E, Nassor MH (2015). Loop-mediated isothermal amplification (LAMP) for point-of-care detection of asymptomatic low-density malaria parasite carriers in Zanzibar. Malar J.

[36] Vallejo AF, Martinez NL, Gonzalez IJ, Arevalo-Herrera M, Herrera S (2015). Evaluation of the loop mediated isothermal DNA amplification (LAMP) kit for malaria diagnosis in *P. vivax* endemic settings of Colombia. PLoS Negl Trop Dis.

[37] Oriero EC, Okebe J, Jacobs J, Van Geertruyden JP, Nwakanma D, D'Alessandro U (2015). Diagnostic performance of a novel loop-mediated isothermal amplification (LAMP) assay targeting the apicoplast genome for malaria diagnosis in a field setting in sub-Saharan Africa. Malar J.

[38] Kugelman JR, Wiley MR, Mate S, Ladner JT, Beitzel B, Fakoli L (2015). Monitoring of Ebola virus Makona evolution through establishment of advanced genomic capability in Liberia. Emerg Infect Dis.

[39] Quick J, Loman NJ, Duraffour S, Simpson JT, Severi E, Cowley L (2016). Real-time, portable genome sequencing for Ebola surveillance. Nature.

[40] Benítez-Páez A, Portune KJ, Sanz Y (2016). Species-level resolution of 16S rRNA gene amplicons sequenced through the MinION™ portable nanopore sequencer. Gigascience..

[41] Elia MIY, Isra W, Mochammad H (2015). The evaluation on molecular techniques of reverse transcription loop-mediated isothermal amplification (RT-LAMP), reverse transcription polymerase chain reaction (RT-PCR), and their diagnostic results on MinION™ nanopore sequencer for the detection of dengue virus serotypes. American Journal of Microbiological Research.

[42] Imwong M, Tanomsing N, Pukrittayakamee S, Day NP, White NJ, Snounou G (2009). Spurious amplification of a *Plasmodium vivax* small-subunit RNA gene by use of primers currently used to detect P. Knowlesi. J Clin Microbiol.

[43] Singh B, Kim Sung L, Matusop A, Radhakrishnan A, Shamsul SS, Cox-Singh J (2004). A large focus of naturally acquired *Plasmodium knowlesi* infections in human beings. Lancet.

[44] Shin J, Lee S, Go MJ, Lee SY, Kim SC, Lee CH (2016). Analysis of the mouse gut microbiome using full-length 16S rRNA amplicon sequencing. Sci Rep.

[45] Deschamps S, Mudge J, Cameron C, Ramaraj T, Anand A, Fengler K (2016). Characterization, correction and *de novo* assembly of an Oxford Nanopore genomic dataset from Agrobacterium tumefaciens. Sci Rep.

[46] Li C, Chng KR, Boey EJ, Ng AH, Wilm A, Nagarajan N (2016). INC-Seq: accurate single molecule reads using nanopore sequencing. Gigascience.

[47] Morris U, Khamis M, Aydin-Schmidt B, Abass AK, Msellem M, Nassor MH (2015). Field deployment of loop-mediated isothermal amplification for centralized mass-screening of asymptomatic malaria in Zanzibar: a pre-elimination setting. Malar J.

[48] Falade CO, Ajayi IO, Nsungwa-Sabiiti J, Siribié M, Diarra A, Sermé L (2016). Malaria rapid diagnostic tests and malaria microscopy for guiding malaria treatment of uncomplicated fevers in Nigeria and prereferral cases in 3 African countries. Clin Infect Dis.

[49] Forney JR, Wongsrichanalai C, Magill AJ, Craig LG, Sirichaisinthop J, Bautista CT (2003). Devices for rapid diagnosis of malaria: evaluation of prototype assays that detect *Plasmodium falciparum* histidine-rich protein 2 and a *Plasmodium vivax*-specific antigen. J Clin Microbiol.

[50] Fernando SD, Karunaweera ND, Fernando WP (2004). Evaluation of a rapid whole blood immunochromatographic assay for the diagnosis of *Plasmodium falciparum* and *Plasmodium vivax* malaria. Ceylon Med J.

[51] Pattanasin S, Proux S, Chompasuk D, Luwiradaj K, Jacquier P, Looareesuwan S (2003). Evaluation of a new plasmodium lactate dehydrogenase assay (OptiMAL-IT) for the detection of malaria. Trans R Soc Trop Med Hyg.

[52] Iqbal J, Muneer A, Khalid N, Ahmed MA. Performance of the OptiMAL test for malaria diagnosis among suspected malaria patients at the rural health centers. Am J Trop Med Hyg. 2003; 68:624-8. 10.4269/ajtmh.2003.68.624 12812358

[53] Farcas GA, Zhong KJ, Lovegrove FE, Graham CM, Kain KC (2003). Evaluation of the Binax NOW ICT test versus polymerase chain reaction and microscopy for the detection of malaria in returned travelers. Am J Trop Med Hyg..

[54] Cao MD, Ganesamoorthy D, Elliott AG, Zhang H, Cooper MA, Coin LJ (2016). Streaming algorithms for identification of pathogens and antibiotic resistance potential from real-time MinION™ sequencing. Gigascience.

[55] Wongsrichanalai C, Barcus MJ, Muth S, Sutamihardja A, Wernsdorfer WH (2007). A review of malaria diagnostic tools: microscopy and rapid diagnostic test (RDT). Am J Trop Med Hyg..

[56] Adams ER, Schoone GJ, Ageed AF, Safi SE, Schallig HD (2010). Development of a reverse transcriptase loop-mediated isothermal amplification (LAMP) assay for the sensitive detection of *Leishmania* parasites in clinical samples. Am J Trop Med Hyg.

[57] Badolo A, Okado K, Guelbeogo WM, Aonuma H, Bando H, Fukumoto S (2012). Development of an allele-specific, loop-mediated, isothermal amplification method (AS-LAMP) to detect the L1014F kdr-w mutation in Anopheles Gambiae s. L. Malar J.

[58] Iwasaki MYT, Otsuka K, Suzuki W, Nagamine K, Hase T, Tatsumi K (2003). Validation of the loop-mediated isothermal amplification method for single nucleotide polymorphism genotyping with whole blood. Genome Letters.

[59] Pan L, Li J, Zhang WN, Dong L (2015). Detection of the I1781L mutation in fenoxaprop-p-ethyl-resistant American sloughgrass (Beckmannia Syzigachne Steud.), based on the loop-mediated isothermal amplification method. Pest Manag Sci.

[60] Itonaga M, Matsuzaki I, Warigaya K, Tamura T, Shimizu Y, Fujimoto M (2016). Novel methodology for rapid detection of KRAS mutation using PNA-LNA mediated loop-mediated isothermal amplification. PLoS One.

[61] Howard RL, French DJ, Richardson JA, O'Neill CE, Andreou MP, Brown T (2015). Rapid detection of diagnostic targets using isothermal amplification and HyBeacon probes--a homogenous system for sequence-specific detection. Mol Cell Probes.

[62] Karamitros T, Magiorkinis G (2015). A novel method for the multiplexed target enrichment of MinION next generation sequencing libraries using PCR-generated baits. Nucleic Acids Res.

